# Genitourinary syndrome: pelvic examination contributes to optimal management in postmenopausal women

**DOI:** 10.61622/rbgo/2026rbgo58

**Published:** 2026-08-20

**Authors:** Carolina Neves Buhl, Carina Cordeiro Nunes, Aline de Bastos Ferreira, Ricardo Ruiz Garcia de Almeida, Adriana Orcesi Pedro, Lucia Helena Simões Costa-Paiva, Gloria Bachmann, Luiz Francisco Baccaro

**Affiliations:** 1 Universidade Estadual de Campinas Department of Tocogynecology Campinas SP Brazil Department of Tocogynecology, Universidade Estadual de Campinas, Campinas, SP, Brazil.; 2 Rutgers Robert Wood Johnson Medical School Brunswick NJ United States of America Rutgers Robert Wood Johnson Medical School, Brunswick, NJ, United States of America.

**Keywords:** Climacteric, Atrophic vaginitis, Menopause, Postmenopausal, Genitourinary syndrome, Pelvic examination

## Abstract

**Objective:**

This cross-sectional study aimed to evaluate the correlation between self-reported symptoms, clinical assessment and vaginal hormonal cytology in diagnosing genital atrophy in postmenopausal women attending a tertiary menopause clinic.

**Methods:**

A convenience sample of postmenopausal women aged 30 years and older, experiencing a broad spectrum of climacteric symptoms and without prior treatment or debilitating conditions, was included in the study. The study protocol encompassed: 1) Menopause Rating Scale (MRS), 2) pelvic examination using the Vaginal Health Index (VHI), and 3) assessment of vaginal hormonal cytology through the Meisels Index (MI). Univariate and bivariate descriptive analyses, as well as simple and multiple linear regression analyses, were conducted to identify factors associated with MI, with a significance level set at 5%.

**Results:**

One hundred and fifty-four women were studied, with a mean age of 50 years (±9) and mean age at menopause onset of 44.3 years (±6). The mean body mass index (BMI) was 28.7 (±5.4). The average MI was 47.8 (±23.8), MRS urogenital domain score was 6.1 (±3.7), and VHI score was 16.7 (±3.8). Multiple regression analysis showed significant associations between MI and total VHI (p < 0.001), BMI (p = 0.0263), and age (p = 0.0004). Women with higher MI tended to have higher VHI scores, higher BMI, and were younger.

**Conclusion:**

The study suggests that pelvic examination contributes to optimal management of all postmenopausal women, including those who do not report symptoms.

## Introduction

The climacteric encompasses physiological changes due to ovarian function decline, preceding and following the final menstrual period.^(1)^ In Brazil, the average natural menopausal age is 51.2 years, and data suggest that it affects a substantial portion of women's lives due to climacteric symptoms.^(2)^

Genitourinary syndrome of menopause (GSM) is defined as a set of signs and symptoms resulting from estrogen deficiency in the female genitourinary tract - vagina, vulva, urethra, and bladder - leading to histological and anatomical changes.^(3)^ Genital symptoms (dryness, burning, and irritation), urinary symptoms (dysuria, nocturia, urgency, recurrent urinary tract infections), and sexual symptoms (dyspareunia and lack of lubrication) may be present. They can be mild, moderate, or severe due to vaginal elasticity reduction, increased vaginal pH, changes in vaginal flora, and decreased lubrication.^(3)^

Diagnosis is clinical, through patient report and gynecological examination.^(4)^ Complementary evaluations may include vaginal pH measurement and application of the Vaginal Health Index (a scale of vaginal assessment parameters graded from 1 to 5, including elasticity, fluid volume, pH, epithelial integrity, and moisture).^(5)^ Vaginal smear cytology for hormonal evaluation, along with the vaginal maturation index (which assesses the relationship between superficial, intermediate, and deep cells through the Meisels index) represent objective parameters for assessing genital atrophy.^(6)^

GSM prevalence ranges from 36% to almost 90% among postmenopausal women.^(7)^ Unlike other climateric symptoms that are temporary and tend to diminish over time, GSM symptoms tend to worsen with age if not treated.^(8)^ In addition to potentially presenting exuberant symptoms, GSM is clinically important due to the changes it may cause in cervical cytology, as well as the increased susceptibility to acquiring sexually transmitted infections (STIs) due to the thinned and less elastic vaginal epithelium, making early diagnosis and treatment even more crucial.^(9)^

In Brazil, 44% of women believe that vaginal symptoms affect their self-esteem.^(10)^ Women who are not sexually active may experience genital discomfort as well. Despite this high prevalence, more than 70% of symptomatic women do not report their symptoms to their clinician and many clinicians do not consistently inquire about GSM, which results in treatment not routinely prescribed for these women.^(10, 11)^ The gynecological physical examination has the potential to induce discomfort in some women, with descriptions of feelings such as unease, embarrassment, and fear due to the invasive nature of the procedure.^(12, 13)^ The perception of discomfort varies among women and can be influenced by several factors. The correlation between symptoms and vaginal physical changes is not simple or exact, and still requires further studies.^(9)^

GSM is often underdiagnosed, as many women fail to report their symptoms. It significantly it affects the quality of life of postmenopausal women and occurs in a population for whom pelvic examination may be uncomfortable. This study aimed to correlate symptoms, clinical findings, and vaginal hormonal cytology in diagnosing genital atrophy among women attending a tertiary menopause clinic. It also sought to determine whether performing gynecological examinations is justified in postmenopausal women, including those without symptoms or indications for colpocytological screening.

## Methods

This cross-sectional study was conducted at the menopause outpatient clinic of a tertiary hospital affiliated to the Department of Obstetrics and Gynecology, Faculty of Medical Sciences, State University of Campinas from November 2022 to November 2023. Inclusion criteria were women over 30 years experiencing menstrual irregularity, climateric symptoms or urogenital complaints, who started follow-up during the study period. We acknowledge that hypoestrogenism and climacteric symptoms occurring before the age of 40 are classically defined as premature ovarian insufficiency (POI). However, in the present study, women aged 30 years and older were included because this is a tertiary referral center that follows a significant number of patients with treatment-induced menopause secondary to oncologic therapies. This population represents an important subgroup of women affected by genitourinary syndrome of menopause and was therefore of particular interest for this investigation. Cases of spontaneous premature ovarian insufficiency or those unrelated to cancer treatment were not included. Exclusion criteria included (1) current or recent (< 6 months) treatment for climacteric symptoms, (2) severe or debilitating health condition, such as inability to communicate or movement and (3) premature ovarian failure due to causes other than cancer treatment.

This study forms part of a broader research titled "Menopause in Women with a History of Malignant Gynecological Neoplasms", which will address various aspects of climacteric in patients with and without a history of treatment for gynecological neoplasia. Data collection included sociodemographic variables, clinical characteristics, the Menopause Rating Scale (MRS) for symptom assessment, general physical examination data (e.g., BMI), and gynecological assessment using the Vaginal Health Index (VHI) and vaginal smear cytology.^(5,6,14)^

Meisels Index (calculated from the cell maturation index obtained through manual analysis of vaginal smear cytology under a microscope): a continuous quantitative variable. Possible values range from 0 to 100, where higher values indicate a more eutrophic epithelium, and lower values indicate a more atrophic epithelium. The index is calculated using the formula: [parabasal cells × 0] + [intermediate cells × 0.5] + [superficial cells × 1].^(6)^

Clinical and sociodemographic characteristics of patients were collected, including age, age at menopause, type of menopause (natural or induced by surgery, chemotherapy or radiotherapy), education level, marital status, body mass index (BMI), race, sexual activity, history of hypertension and diabetes mellitus, smoking and history of treatment for gynecological neoplasia (including breast, ovarian, endometrial and cervical cancer). Menopause rating scale questionnaire: eleven questions which consisted of symptoms to be evaluated graded from 0 to 4, with a total score range 0 to 44. In this study it was considered for analysis MRS sum of questions 8 (sexual problems - changes in desire, activity, and sexual satisfaction), 9 (urinary problems - incontinence, increased frequency, retention), and 10 (vaginal dryness, burning sensation, difficulty in sexual intercourse) – regarding the genitourinary domain, and question 10 individually. Each question could be answered from 0 to 4, with higher values corresponding to more intense symptoms.^(14,15)^ The authors have permission to use this instrument from the copyright holders, and the translated questionnaire was obtained through the developers themselves. Vaginal evaluation (VHI) was also performed. Parameters evaluated include elasticity, fluid volume, pH, integrity of the epithelium and vaginal moisture. The sum of parameters evaluated yields results between 5 and 25, where a lower value indicates a more atrophic epithelium, and a higher value indicates a more eutrophic epithelium. The cutoff point for considering atrophic epithelium were values above 15.^(5,16)^ The authors have permission to use this instrument from the copyright holders.

Statistical analyses included descriptive statistics, Mann-Whitney and Kruskal-Wallis tests, Spearman's correlation and simple and multiple linear regression (Stepwise selection) to identify factors associated with the Meisels Index.^(17, 18)^ Data were analyzed using SAS System for Windows (version 9.4), with a significance level set at p<0.05. This manuscript was prepared in accordance with the STROBE (Strengthening the Reporting of Observational Studies in Epidemiology) guidelines for cross-sectional studies to ensure transparency and completeness in the reporting of observational research.^(19)^

Approval was obtained from the Unicamp Research Ethics Committee through the Platform Brasil (CAE: 59356022.0.0000.5404) 5.658.032, and all participants provided written informed consent. Following the inclusion and exclusion criteria, 182 women were invited to participate in the study, and a total of 154 women accepted participation.

## Results

Data from 154 postmenopausal women were obtained, with a mean age of 50 years (± 9), and mean age at menopause of 44.3 years (± 6). Of all participants, 50.6% (78) had a history of treatment for gynecological malignancy, including breast cancer, ovarian cancer, endometrial cancer, and cervical cancer. Induced menopause occurred in 56.6% (86) of cases, either post-chemotherapy, post-radiotherapy or surgical due to benign or malignant causes. The majority of women had a stable partnership (63%, 97), with 65.6% (101) being sexually active. The mean BMI was 28.7 (± 5.4). The mean Meisels index was 47.8 (± 23.8). The mean score for the urogenital domain of the Menopause Rating Scale (considering the sum of questions 8, 9 and 10) was 6.1 (± 3.7), while the mean score for question 10 singly was 2.2 (± 1.7). The mean VHI score was 16.7 (± 3.8). Regarding age, 50% (77) of women were between 44 years (1st quartile) and 55 years (3rd quartile) at their first consultation. However, the standard deviation of 9.3 indicates a high dispersion in women's ages. Similarly, 50% (77) of observations of age at menopause onset were between 41 years (1st quartile) and 49 years (3rd quartile), indicating concentration of menopause onset between ages 40 and 50 years. Concerning education level, table 1a shows that 38.4% had completed elementary school, 41% had completed high school, and 20.6% had a college degree. regarding race/ethnicity, table 1a indicates that 62.3% of women self-identified as white, 28% as mixed race, and 9.7% as black. regarding comorbidities, 35.7% of evaluated women had hypertension, 9% were diabetic, and 16.9% were smokers, as shown in table 1b.

**Table 1a:** Descriptive analysis of categorical variables - sociodemographic characteristics

Variable		n(%)
Age		
	30-39	15(9.74)
	40-49	59(38.31)
	50-59	60(38.96)
	60-69	15(9.74)
	70-79	4(2.60)
	≥ 80	1(0.65)
Education level		
	Elementary	58(37.66)
	High School	62(40.25)
	Higher	31(20.12)
	Missing	3(1.97)
Marital status		
	Fixed partner	97(62.99)
	Separeted/Single	57(37.01)
Race/color		
	White	96(62.34)
	Black	15(9.74)
	Brown	43(27.92)
Cancer		
	Yes	76(49.35)
	No	78(50.65)
Parity		
	Nulliparous	25(16.23)
	Primiparous	31(20.13)
	Multiparous	98(63.64)
Menopause		
	Natural	66(43.42)
	Induced	86(56.58)

**Table 1b:** Descriptive analysis of categorical variables - clinical characteristics

Variable		n(%)
Sexual activity		
	Yes	101(65.58)
	No	53(34.42)
Arterial hypertension		
	Yes	55(35.71)
	No	99(64.29)
Diabetes		
	Yes	14(9.09)
	No	140(90.91)
Smoking		
	Yes	26(16.88)
	No	128(83.12)
Alcoholism		
	Yes	5(3.25)
	No	149(96.75)
MRS10		
	0	55(35.71)
	1	22(14.29)
	2	26(16.88)
	3	27(17.53)
	4	22(14.29)
	Missing	2(1.30)
VHI Score		
	< 15	50(32.47)
	≥ 15	81(52.60)
	Missing	23(14.93)

After completing the descriptive analysis, a comparative analysis of the Meisels index and other variables of interest was conducted. Significant differences in the Meisels index were observed in table 2 based on sexual activity (lower values in those without activity, p-value 0.048) and total VHIS (lower values in those with total VHIS ≤15, the cutoff point for an atrophic epithelium, p-value <0.001). Marital status, race/color, history of cancer, parity and type of menopause had no association with the Meisels index. Table 3 presents the correlations between numerical variables and the Meisels index. A statistically significant association can be observed between women's age and age at menopause and MI (inversely correlated, p-value 0.0268), BMI and MI ((p-value 0.0263) and VHIS and MI (p-value <0.001), both directly correlated. Younger women have better MI, as do women with higher BMI and higher VHIS. The intensity of symptoms reported by women was not associated with MI, whether in the assessment of MRS 10 singly (p-value 0.7292) or in the MRS Score 8+9+10 (p-value 0.5705).

**Table 2:** Meisels index and categorical variables

Variables		Mean	SD	Minimum	Q1	Median	Q3	Maximum	p-value
Marital status									0.18**
	Married	46.8	22.9	0	30	55	60	90	
	Separate	49.4	23.9	5	30	55	65	90	
	Single	54	24.3	0	40	55	70	85	
	Widow	30	31.1	0	0	30	60	60	
Race/color									0.45**
	White	47.3	23.7	0	40	55	60	85	
	Black	44	25.5	0	30	55	55	80	
	Brown	50	23.9	0	30	55	65	90	
History of cancer									0.12*
	No	50.4	23.4	0	50	55	65	90	
	Yes	45	24.2	0	30	55	60	90	
Parity									0.58**
	Nulliparous	43.7	26.1	0	25	55	55	85	
	Primiparous	48.1	22.1	0	40	55	60	85	
	Multiparous	48.6	24	0	35	55	65	90	
Menopause									0.16*
	Natural	43.8	25.3	0	20	55	60	85	
	Induced	50.6	22.7	0	40	55	65	90	
Sexual activity									0.04*
	Yes	50.9	22.2	0	40	55	65	90	
	No	41.3	26.1	0	15	55	60	85	
VHI score									<0.01*
	<=15	37.6	25.4	0	10	45	60	80	
	>15	54.3	19.8	5	50	55	65	90	

* Mann-Whitney test

** Kruskal-Wallis test

**Table 3:** Correlation between numerical variables and the Meisels Index

Variables	Spearman's correlation coefficient (r)	p-value*
Age	-0.29305	0.0004
Age of menopause	-0.18780	0.0268
BMI	0.18645	0.0263
MRS10	0.02973	0.7292
MRS score (8+9+10)	0.04818	0.5705
VHI score	0.35303	<0.0001

* p<0.05 considered significant

The results of the final statistical model (multiple linear regression analyses, using Stepwise variable selection criteria) to assess factors associated with the Meisels index founded a significant relationship between total VHIS (p-value <0.001), BMI (p-value 0.017), age (p-value 0.039) and the Meisels index. Women with higher Meisels indices were those with higher total VHIS, BMI, and younger age.

## Discussion

A convenience sample of women experiencing a broad spectrum of climacteric symptoms was included in the study. Assessment of the vaginal epithelium using the Meisels Index showed a statistically significant association with sexual activity, VHIS, age, and BMI. Higher Meisels Index values, suggestive of a more trophic epithelium, were observed among sexually active women, those with better vaginal health in physical exam (VHIS >15), higher BMI, and younger age. No relevant associations were identified between the Meisels Index and marital status, race/ethnicity, cancer history, parity, or type of menopause.

When comparing GSM treatments, including moisturizers and/or placebo, the data would be more comprehensive if cytological parameters, pH, or vaginal flora were included.^(20,21)^ However, Davila et al.^(22)^ conducted a cross-sectional study with 135 volunteer women, evaluating the presence and severity of vaginal atrophy symptoms and mucosal changes through pelvic examination. They correlated clinical manifestations, symptoms, pH, and vaginal cytological maturation value with the purpose of determining the degree of correlation between physical examination and symptoms. Their data did not show a correlation between symptoms of dryness, pain, vaginal irritation, and dyspareunia and low epithelial maturation value. They suggested that isolated symptoms should not be used as a guide for atrophy treatment. Furthermore, the study by Greendale et al.^(23)^ assessed the reproducibility of findings in vaginal examination representing genital atrophy from a cross-section of 40 women undergoing gynecological physical examination with specific criteria. The study demonstrated that decreased elasticity, roughness, and the presence of petechiae were significantly associated with low epithelial maturation, corroborating the importance of physical examination for this diagnosis.

Hormonal changes at menopause induce alterations in vaginal bacterial flora.^(24)^ After menopause, the vagina is colonized predominantly by fecal-type flora, while lactobacilli predominate during the fertile period of life. Anaerobic flora may contribute to symptoms of vaginal atrophy. Conversely, hormonal replacement increases the abundance of vaginal lactobacilli that produce lactic acid from glycogen, creating an acidic pH that maintains vaginal health.^(25, 26)^ Decreased estrogen levels diminish the maturation of stratified squamous epithelium, resulting in an epithelium composed of basal and intermediate cells and few superficial cells. There is a relationship between sufficient vaginal estrogen and vaginal microorganisms and their metabolic products. The loss of vaginal acidity makes the vagina more susceptible to pathologic bacteria, including those found in the bowel and skin, sexually transmitted infections, and bacterial vaginosis.^(27)^ In the absence of vaginitis, a high vaginal pH may reflect low levels of circulating estrogen (estradiol levels <40 pg/ml) or inadequate response of the atrophic vaginal epithelium to estrogen therapy.^(26, 28)^ Brizzolara et al.^(29)^ concluded that a vaginal pH above 6.0 indicates high levels of parabasal cells. Other studies have shown that a vaginal pH (without infections) above 5.0 is associated with decreased serum estradiol and menopause.^(30)^ Thus, the evaluation of vaginal pH during gynecological physical examination is a valuable source for assessing GSM.

Aromatase enzyme plays a central role in hormonal synthesis, converting androgens into estrogens, with its activity increased in obese women, mainly due to excess adipose tissue. Peripheral conversion of precursors into estradiol and estrone by adipose tissue results in higher circulating estrogen levels even without hormonal replacement, contributing to better vaginal health.^(31, 32)^ This may explain the finding that body mass index correlates linearly with genital trophism in this study. Regarding higher Meisels index values found in sexually active women, one study observed that genital atrophy is milder in postmenopausal women who maintain sexual activity. Sexual activity with vaginal penetration appears to support vaginal elasticity, flexibility, and lubrication. In general, women who engage in sexual activity report fewer symptoms of atrophic vaginitis and, on vaginal examination, show less evidence of vaginal stenosis and narrowing.^(33)^

Cultural and socioeconomic factors may also influence the perception and reporting of GSM. Women from lower socioeconomic backgrounds or with limited access to healthcare services may underreport symptoms or avoid gynecological examinations due to embarrassment, cultural beliefs, or misinformation. Previous studies have demonstrated that lower educational level, rural residence, and reduced access to health information are associated with higher prevalence and severity of GSM symptoms, as well as poorer self-care capacity and quality of life.^(34, 35)^ However, in the present study, no significant association was found between the evaluated socioeconomic variables and the Meisels Index. This finding may be partly explained by the study setting—a single tertiary center of the public healthcare system—where the sample represents a more homogeneous population in terms of socioeconomic background and access to medical care. These aspects suggest that, in this context, biological factors may play a more prominent role than sociodemographic determinants in the severity of vaginal atrophy.

It is known that oncological treatments also contribute to symptoms of genital atrophy, due to hypoestrogenism resulting from it, which could exacerbate GSM symptoms.^(36, 37)^ Although a significant portion of the studied population had a history of gynecological cancer (50.6%), having undergone treatments such as surgery, chemotherapy, radiotherapy, or antiestrogen therapy, there was no significant difference in the Meisels index between these patients and those without a history of cancer. Further studies are needed to understand the intensity of these symptoms and cytological changes in this population, and its needs for individualized care.

This study has limitations, including its cross-sectional design, use of a convenience sample and inclusion of women from a single tertiary center, which may restrict generalizability. The cross-sectional design precludes establishing cause-and-effect relationships. The inclusion of women with a history of treatment for gynecological neoplasia may have influenced a lower average age of menopause than that found in the Brazilian population.^(2)^ The use of a convenience sample may have resulted in certain variables being underrepresented in statistical analysis, limiting the generalizability of the findings to the broader population. Moreover, the sample's restriction to women attending a menopause clinic introduces selection bias, as these individuals may differ from those who do not seek treatment or receive care elsewhere. Consequently, the conclusions drawn are primarily applicable to this specific population and may not fully capture the diversity of experiences and manifestations of GSM in broader healthcare settings. Future research in this field should ideally include a more diverse group of women from various backgrounds and centers to provide a more comprehensive understanding of genital atrophy and its correlation with climacteric symptoms. However, standardized assessment tools and consistent clinical evaluation strengthen its internal validity, ensures methodological rigor and facilitates reproducibility in other clinical settings.

## Conclusion

The findings suggest that urogenital symptoms reported by women not always correlate with hormonal cytology results. Factors associated with estrogen loss were age, BMI, and signs of atrophy at pelvic examination. Pelvic examination remains valuable for diagnosing and optimizing management of GSM, even in women who do not report urogenital symptoms. Clinicians should routinely inquire about GSM symptoms and perform gynecological evaluation. Future studies should explore the influence of cultural and oncological factors and evaluate long-term outcomes of different management strategies for GSM.

## Data Availability

The data that support the findings of this study are openly available in Unicamp Research Data Repository (v1) at doi:10.25824/redu/N5RBUT.
